# Olfactory and gustatory disorders in COVID-19

**DOI:** 10.1007/s40629-022-00216-7

**Published:** 2022-06-20

**Authors:** Ludger Klimek, Jan Hagemann, Julia Döge, Laura Freudelsperger, Mandy Cuevas, Felix Klimek, Thomas Hummel

**Affiliations:** 1 Center for Rhinology and Allergology, Wiesbaden, Germany; 2 Universitätsmedizin Mainz, Department of Otolaryngology, Head and Neck Surgery, Langenbeck Str. 1, 55101 Mainz, Germany; 3 Universitätsklinikum der Carl Gustav Carus-Universität Dresden, Department of Otolaryngology, Head and Neck Surgery, TU Dresden, Dresden, Germany

**Keywords:** Anosmia, COVID-19, Postviral anosmia, Rhinitis, Olfactory dysfunction

## Abstract

Loss of olfaction is one of the symptoms most commonly reported by patients with coronavirus disease 2019 (COVID-19). Although the spontaneous recovery rate is high, recent studies have shown that up to 7% of patients remain anosmic for more than 12 months after the onset of infection, leaving millions of people worldwide suffering from severe olfactory impairment. Olfactory training remains the first recommended treatment. With the continued lack of approved drug treatments, new therapeutic options are being explored. This article reviews the current state of science on COVID-19-related olfactory disorders, focusing on epidemiology, pathophysiology, cure rates, currently available treatment options, and research on new treatments.

## Introduction

In addition to sinunasal diseases and age-related olfactory disorders, postviral anosmia is a major cause of olfactory disorders in adults [1–6].

Classically, the specialties of otolaryngology and neurology deal with olfactory dysfunction. However, the high prevalence of olfactory dysfunction (OD) in SARS-CoV‑2 infected individuals and its importance as a diagnostic marker have made olfactory investigations a focus of interest in almost all other specialties as well.

Below, we provide an overview of olfactory disorders in the context of COVID-19 disorders, underlying pathophysiology, cure rates, and potential therapeutic options.

## Prevalence and presentation of olfactory loss in COVID-19

Severe acute respiratory syndrome coronavirus 2 (SARS-CoV-2) causes respiratory and general illness with coronavirus disease 2019 (COVID-19) [7–10]. The COVID-19 pandemic is the most serious public health crisis of this decade, with nearly 300 million people sickened worldwide and approximately 5.5 million deaths (www.worldometers.info/coronavirus/ [as of January 7, 2022]). Early in the pandemic, loss of sense of smell was described as a potential marker for COVID-19 in March 2020 [11]. There is now ample evidence that olfactory dysfunction is one of the most common symptoms of COVID-19 infection. A meta-analysis of 3563 patients published in May 2020 found a median prevalence of self-reported olfactory loss of 47% (95% confidence interval 36–59%), ranging from 11–84% in the included case series [12]. It should be noted that regional differences on the prevalence of OD are also likely, at least study data from different continents (Asia, North America, Europe) suggest this [13–15]. Moreover, comparability between studies with and without psychophysical olfactory testing is severely limited because patients’ self-assessment of OD underestimates the true prevalence compared to measurements of olfactory function with psychophysical testing procedures. For example, in 60 hospitalized study participants, Moein et al. demonstrated that 98% had some degree of OD on psychophysical testing, whereas only 35% of participants actively reported loss of olfactory function [16]. In contrast, assessment with psychophysical tests at the time of disease alone may overestimate the prevalence of COVID-19-related olfactory loss because it includes all those patients who were unaware of a pre-existing olfactory disorder prior to their COVID-19 disease [17]. A systematic review suggests that olfactory dysfunction is reported less frequently with increasing age in COVID-19 [18], which has been attributed to age-dependent decreased expression of angiotensin-converting enzyme 2 (ACE2) receptors in the olfactory epithelium [19], but may also reflect the age-related increasing background prevalence of olfactory dysfunction.

Olfactory dysfunction may be the only symptom of disease in patients with COVID-19 [11], and in 23% (95% CI 13–29%) of reported cases included in a May 2020 systematic review, it temporally preceded other symptoms and appeared to be more common in women [20]. A French study [21] of 114 patients with confirmed COVID-19 infection found loss of smell in 47% of patients, but less than 5% of patients had other nasal symptoms such as rhinorrhea and nasal congestion. Other studies [22] have also found that patients with COVID-19-related anosmia do not report rhinitis symptoms otherwise typically associated with viral respiratory infection. The mixed symptom picture of SARS-CoV‑2 infections has been under continuous surveillance since the onset of the pandemic, with recent use of app-based methods (“ZOE symptom app”) with large numbers of study participants [23]. These and other recent data suggest substantial variation in the prevalence of olfactory dysfunction among different viral variants, with very different rates reported in different geographic regions worldwide [24], as well as different rates at different times during the pandemic [25]. It should be noted that, to date, no significant change in the composition of COVID-19 symptoms has been observed with vaccination either, and thus vaccination cannot be assumed to protect against OD [26, 27].

It has been postulated that olfactory impairment may have prognostic value in predicting the severity of COVID-19. An early study by Yan et al. [14] suggested that loss of olfaction was slightly more often associated with milder disease that did not require hospitalization. Inpatients were significantly less likely to report anosmia/hyposmia than outpatients (27% vs. 67%, *p* < 0.001) [14]. This is consistent with systematic reviews that found the prevalence of self-reported olfactory loss to be highly dependent on the patient’s clinical situation. In hospitalized patients, the overall prevalence was 31%, but increased to 67% in mild-to-moderate symptomatic patients isolated at home [12]. However, the lack of standardized populations and olfactory tests could bias the results of such studies. In contrast, a prospective study of 106 patients [28] found no association between olfactory function in the first week of infection and severity of illness. However, in another study [29], the same research group showed no association between viral load and severity of olfactory loss, and no significant statistical correlation between olfactory loss at the onset of infection and severity of chest CT findings [30].

## Pathophysiological mechanisms of olfactory dysfunction in COVID-19

Despite a growing number of studies, the underlying pathophysiological mechanism of anosmia in COVID-19 remains unclear. However, there is much to suggest that SARS-CoV‑2 attacks supporting cells and then leads to secondary damage of olfactory cells [31]. Other mechanisms discussed include edema of the olfactory bulb with impaired transmission, direct damage to the olfactory epithelium (OE), and injury to the olfactory bulb (OB).

For postviral olfactory dysfunction, it is known that complex mechanisms may be causative and are related to a combination of viral load and host immune response, with damage occurring at different levels: olfactory neuroepithelium, olfactory bulb, or other central nervous olfactory centers [32]. SARS-CoV‑2 can enter the central nervous system (CNS) from the peripheral nervous system through different pathways [13, 33], mainly either as direct passage of the virus across the axons of olfactory receptor cells from the olfactory epithelium to the bulbus olfactorius via the lamina cribrosa of the ethmoid bone, or hematogenously or lymphatically by crossing the blood–brain barrier [34].

Local obstruction caused by edema within the olfactory cleft may contribute to early olfactory dysfunction and limit the delivery of odorants to the OE, although nasal obstruction has been reported less frequently in COVID-19 compared with other endemic coronaviruses [35, 36]. Nevertheless, in one study [37], 19 of 20 patients were found to have olfactory fissure obstruction on MRI examination performed within 15 days of the onset of COVID-19-related OD; this was found in only 3 of 19 patients in other radiologic studies in patients with prolonged loss [15].

Damage to the olfactory epithelium has already been demonstrated in postviral olfactory loss [38]. Postmortem studies of COVID-19 patients with anosmia showed focal atrophy of the OE, leukocytic infiltration of the lamina propria, and evidence of axonal damage to olfactory nerve fibers [39]. Animal models of SARS-CoV‑2 [40] showed massive destruction of the olfactory epithelium after nasal viral inoculation and loss of cilia. ACE2 receptors, which are important for SARS-CoV‑2 entry, are expressed by the supporting cells and possibly the horizontal basal cells of the OE [33, 41]. Damage to these cells may result in decreased sensitivity and loss of olfactory receptor neuron (ORN) cilia with loss of smell, even though the ORNs themselves do not express ACE2 receptors and are not directly infected according to previous findings [42].

This hypothesis is consistent with the pattern of early recovery, as direct ORN injury would require a much longer period to achieve OD healing. However, recent in vivo studies in which brush swabs were taken from the olfactory mucosa indicate that both mature sensory neurons and supporting cells appear to be infected [43].

Some recent studies suggest an inflammation-induced loss of olfactory receptor expression on otherwise intact ORN; this is supported by animal models and in olfactory epithelial biopsies taken from COVID-19 patients postmortem. A study of SARS-CoV‑2 in hamsters has shown that the local immune response increases macrophage expression in the OE and lamina propria, which may prevent recovery of the olfactory epithelium and restoration of ORNs [40]. An in vivo study of patients with persistent loss demonstrated viral persistence in the olfactory epithelium associated with progressive inflammation, increased interleukin 6 (IL6), and apoptosis [43]. The regenerative capacity of basal stem cells has been shown to be impaired by inflammation, and this mechanism may therefore be responsible for the persistent olfactory dysfunction [44]. Anecdotal reports of improved olfactory recovery after vaccination may reflect more effective viral clearance [45].

The spread of viruses by retrograde axonal transport to the olfactory bulb (OB) and CNS has been well described [46]. In animal models of OC43 coronavirus infection, virus particles were detected in the OB 3 days after inoculation and in the cortex on day 7 [47]. ACE2 transgenic mice inoculated with SARS-CoV‑1 were also shown to be invaded by the virus with rapid CNS invasion [48]; a similarly high viral load was found along the entire route from the olfactory epithelium to the olfactory bulb [43]. Several case reports documented hyperintensity in the olfactory bulb, which resolved on re-imaging one month later and was accompanied by loss of olfactory bulb volume [49–51]. Signal abnormalities of the olfactory bulb were noted in 19% of 37 COVID-19 patients [52]. One patient with persistent COVID-19-induced OD underwent MRI imaging prior to COVID-19 infection, which provided baseline volumes and confirmed significant atrophy of the OB in images obtained 2 months after disease onset [53]. PET imaging revealed a hypometabolic state in the gyrus rectus in 2 patients with persistent COVID-19 OD [54]. Although evidence of neurotropism, atrophy, and hypometabolism was found in these studies, this could be an indirect consequence of loss of function at the level of the OE. The findings are not direct evidence for retrograde transport of SARS-CoV‑2 into the OB in humans, which is largely considered unlikely [31, 34]. Progress in understanding the mechanism of olfactory loss will contribute to the development of therapeutic options. Therefore, further research in this area is essential.

## Smell tests during the COVID-19 pandemic

Under normal circumstances, psychophysical olfactory and gustatory tests are performed under the guidance of experienced medical personnel (nurses, medical assistants, laboratory personnel) and require personal guidance and supervision. However, during the COVID-19 pandemic, contact avoidance is the order of the day [9, 55, 56].

Telemedicine consultations allow safe testing for patients and staff [57–59] and early detection and monitoring of overall disease progression, including during the infection phase, and thus may be a useful tool for ongoing COVID-19 odor research.

## Recovery of olfactory loss after COVID-19

Numerous studies have since been conducted to assess recovery rates and risk factors for persistence using questionnaires or psychophysical olfactory testing. Early reports indicated very high rates of rapid recovery [60], with many self-assessments indicating complete recovery within an average duration of olfactory loss of 10 days [61]; recovery rates in studies using self-assessments [62–65] range from 32 to 89% [42]. Of interest here is the discrepancy between self-assessment of olfactory function and olfactory function measured in psychophysical tests [62]. A number of studies have now published results at 6 months and beyond. Leedman et al. [66] reported that in a consecutive series of 56 patients with proven COVID-19, 64% were normosmic, 4% were anosmic, and 32% were hyposmic at 6 months, based on evaluation with UPSIT tests. In a case–control study of 100 patients with a median follow-up of 401 days after infection, olfactory dysfunction was found in 46% of affected and 10% of control subjects [67]. Given the large number of people affected by COVID-19, even with the best of the cure rates reported to date, a substantial number of people worldwide will be left with severe olfactory dysfunction.

## Qualitative olfactory disorder—parosmia and phantosmia

Many patients report the development of parosmia, typically after a period of 2–3 months and often after a period of apparent recovery from a preceding hyposmia [68]. Some patients also initially develop parosmia without initially noticing a loss of smell. Some authors report typical indications of “COVID parosmia,” describing it as extremely unpleasant, with a burnt, chemical-like olfactory sensation, but this differs little phenomenologically from previously described nonCOVID-associated olfactory disorders [42, 69, 70]. Common triggers include coffee, onions, garlic, meat, and citrus fruits, as well as toiletries such as mouthwash or toothpaste [42, 71].

The underlying mechanism of parosmia and phantosmia remains unclear. One theory is that a reduced number of functioning olfactory neurons leads to incomplete encoding of odor-induced information by the OR [72], supported in part by the finding of a reduced number of ORs and a dominance of immature neurons in histopathological examination of the olfactory epithelium of deceased COVID-19 patients, but also in animal experiments (see also [73]). It has also been suggested that parosmia may reflect defective stimulus processing in demyelinated neurons [74], neurons of the olfactory mucosa [75], or a central mechanism [76], with abnormal activity detected on positron emission tomography or functional MRI [72, 77]. Interestingly, parosmia seems to be a good prognostic sign [78, 79].

Treatment recommendations for parosmia and phantosmia are not evidence-based to date, although there are anecdotal reports of the use of anticonvulsants such as gabapentin in severe cases [72]. Parosmias, as well as phantosmias, are typically temporary, i.e., they disappear or decrease in intensity and distress to the patient within 6–18 months [70, 80].

## Evaluation of the disturbance of the sense of taste in COVID-19

Subjective impairment of the sense of taste is also among frequently mentioned symptoms in the context of COVID-19 disease; studies suggest a proportion between 40–50% of sufferers [18, 81]. The proportion of those who considered both olfaction and taste to be impaired is high in this context. This suggests that “taste loss” is often understood to mean a loss of taste richness and aromas. While the actual taste qualities are limited to sweet, sour, salty, bitter, umami, and fatty, the retronasal propagation of flavors in the nose is highly important for the overall perception of taste [82, 83]. Validated, blinded testing of the sense of taste using Taste Strips revealed measurable hypogeusia in only 12% and 26% of study participants (*n* = 93 and *n* = 41, respectively) [84, 85]. The actual impairment of taste qualities seems to be less frequent with an inverse pattern than estimated by the sufferers, in contrast to olfactory dysfunction.

## Current therapeutic options for anosmia and hyposmia after COVID-19

There are few established interventions for postviral olfactory dysfunction and, although a number of studies are being conducted, there is currently very little evidence for treatments, specifically for COVID-19-related olfactory dysfunction. One systematic review [86] included only one eligible randomized controlled trial, but noted 8 registered ongoing studies whose results are not (yet) currently available. The included study [87] provides weak evidence for the effect of intranasal steroids (INCS) and oral steroids (OCS) administered to a group of 18 patients 30 days after onset compared to no treatment, with psychophysical olfactory scores measured at baseline and after 20 and 40 days. More pronounced improvement was found in the active treatment group than in the control group. Larger numbers of participants and longer follow-up are needed before definite recommendations can be made.

A systematic review published in 2019 concluded that topical nasal steroids do not improve olfactory dysfunction in nonchronic rhinosinusitis [88]. In contrast, there is a study demonstrating a benefit of budesonide spray in combination with olfactory training: 44% of patients in the active arm showed improvement compared to 27% under olfactory training (OT) [89]. Given the low risk of harm from topical steroids, they could be considered for patients with persistent OD after COVID-19. However, it seems rather unlikely that steroids administered nasally as a spray will reach the olfactory cleft at all—the nose is known to be a very effective filtering system [90, 91].

In a systematic review specifically addressing the use of oral steroids in postviral OD [92], the authors state that similar benefit is usually achieved with placebo as with oral steroids but still recommend steroid use in special cases.

Another review also concludes that there is weak evidence for the successful use of systemic steroids [88]. Guidelines published to support treatment decisions for COVID-19 odor loss [93] suggest that oral steroids are an option for patients in whom the loss lasts longer than 28 days, but spontaneous healing should be awaited in the first few weeks after recovery begins [42]. In this regard, a period of 30 days after the end of COVID-19 disease seems to be an optimal time [42]. However, the use of systemic steroids in postviral olfactory disorders is generally viewed skeptically [94].

There is evidence that olfactory training improves olfactory function in patients with postviral OD. As part of such olfactory training, patients with olfactory dysfunction should smell four powerful odors (olfactory and trigeminal stimuli) for 20–30 s each morning and evening over the course of 3–12 months. A 2016 meta-analysis [95] of all etiologies of OD concluded that olfactory training produced statistically significant improvement in odor discrimination and identification but not olfactory thresholds, although subgroup analysis was worse for patients with postviral OD than for other causes of OD. A 2017 meta-analysis [96] included 6 studies and 455 patients with postviral OD and reported that identification, discrimination, and olfactory threshold improved significantly. A prospective, single-blinded study [97] included 70 patients with postviral OD and controlled patients for 5 months. Forty-five percent of patients with postviral OD achieved significant and clinically meaningful improvement in psychophysically measured olfactory function. Evidence suggests that longer training, changing olfactory agents every 12 weeks, and higher olfactory agent concentrations lead to better outcomes. Most included studies lacked control groups, so spontaneous recovery contributing to the benefits shown cannot be ruled out (but see [98]). However, in the absence of adverse effects, all guidelines recommend that patients undergo olfactory training.

There is limited evidence from nonrandomized trials of other treatments used for a variety of causes of OD, including topical vitamin A, ω‑3 fatty acids, α‑lipoic acid, theophylline, and sodium citrate, but these have not yet received recommendations for use in post-COVID-19 anosmia. Drug therapies based on herbal substances have also been discussed but have not been adequately established to date [99].

Certainly, the COVID-19 pandemic has raised awareness of the impact of olfactory loss and stimulated research into new treatment options. Guidelines and systematic reviews need to be updated regularly to capture new evidence.

## Potential future therapeutic approaches for post-COVID-19 anosmia

Since activation of olfactory mucosa stem cells may be suppressed in the setting of SARS-CoV‑2 infection, stimulation of these cells may promote healing. Platelet-rich plasma (PRP) is known to have anti-inflammatory and regenerative properties, which include upregulation of growth factors such as transforming growth factor, vascular endothelial growth factor, epidermal growth factor, and insulin-like growth factor, and may have neurodegenerative effects. A small pilot study [100] investigated the efficacy of PRP injection into the olfactory cleft in 7 patients. After the 1st and 3rd month, 2 patients with anosmia had no improvement, and 5 patients with hyposmia showed improvement in Sniffin’-Sticks scores [100]. A study of COVID-19 patients is currently underway [42]. Another approach may be mucosal transplants from the olfactory cleft to deliver stem cells [101]. In a study of transgenic mice, the 30-day survival rate in the olfactory bulb was 83% (= 5 of 6 grafts). Histological examination showed that cells resembling olfactory cells developed [102]. In another study in knock-out mice, hyposmia improved after intranasal infusion of purified tissue-specific stem cells. Graft-derived olfactory neuron clusters were confirmed throughout the olfactory epithelium (5 clusters/section, *n* = 6 mice), and functional improvements were measured 3 weeks postinfusion using electrophysiology and behavioral tests [103].

Another approach could be the development of olfactory implants [104]. In a pilot project with 5 patients, an attempt was made to stimulate the olfactory bulb [105]. In 3 of 5 patients, olfactory perception was subjectively reported, but this was not objectively controlled.

## Outlook

Since the total number of patients infected with SARS-CoV‑2 is more than 500 million worldwide at the time of this review, an estimated 18–30 million people suffer from a post-COVD-19 smell disorder, the long-term prognosis of which cannot yet be conclusively assessed. To date, vaccination or other interventions have not been successful in directly influencing the risk for such disorders. Improvements in olfactory dysfunction up to *restitutio ad integrum* are possible, but not all patients are successful with measures such as olfactory training. Studies on prevention and therapy of COVID-associated olfactory dysfunction need to be intensified.
